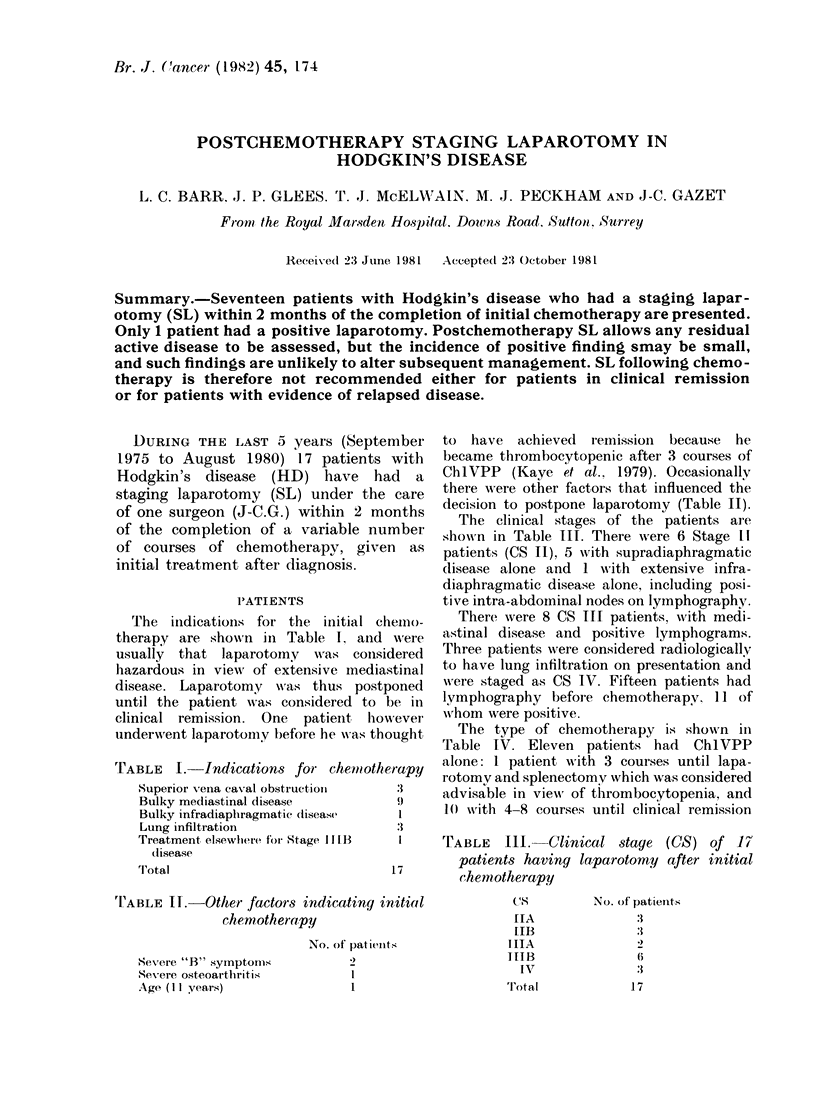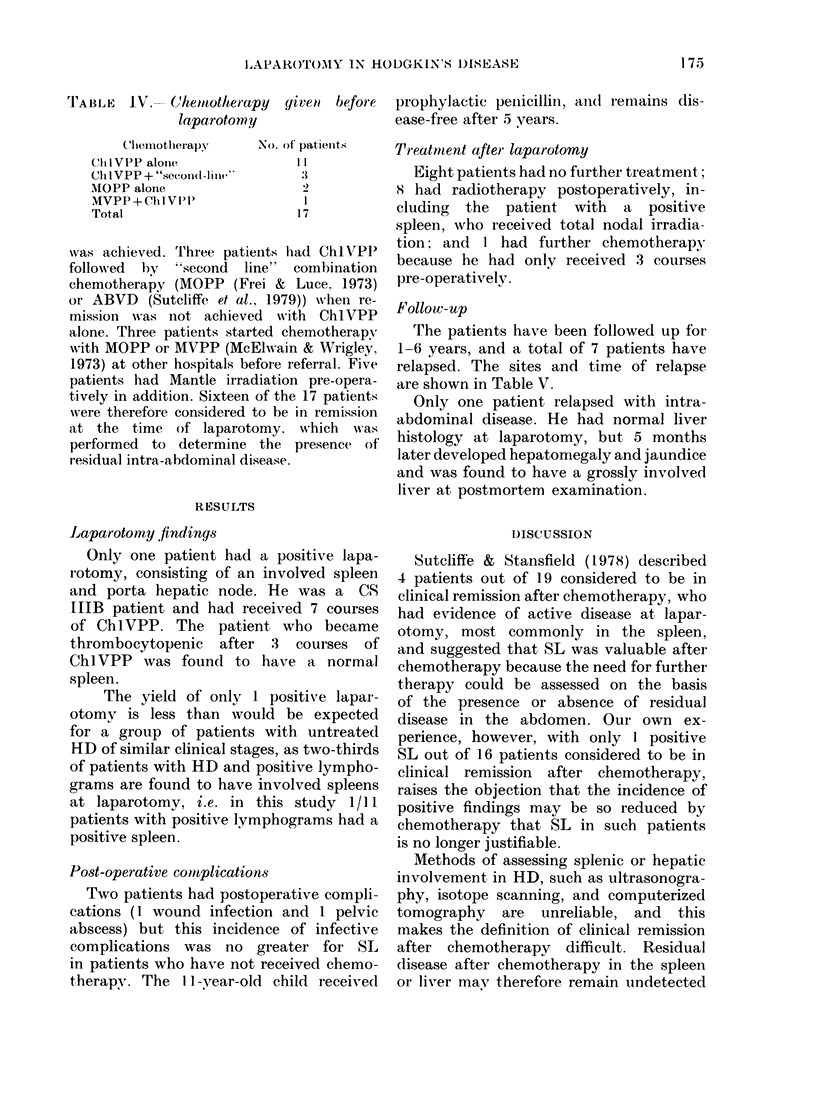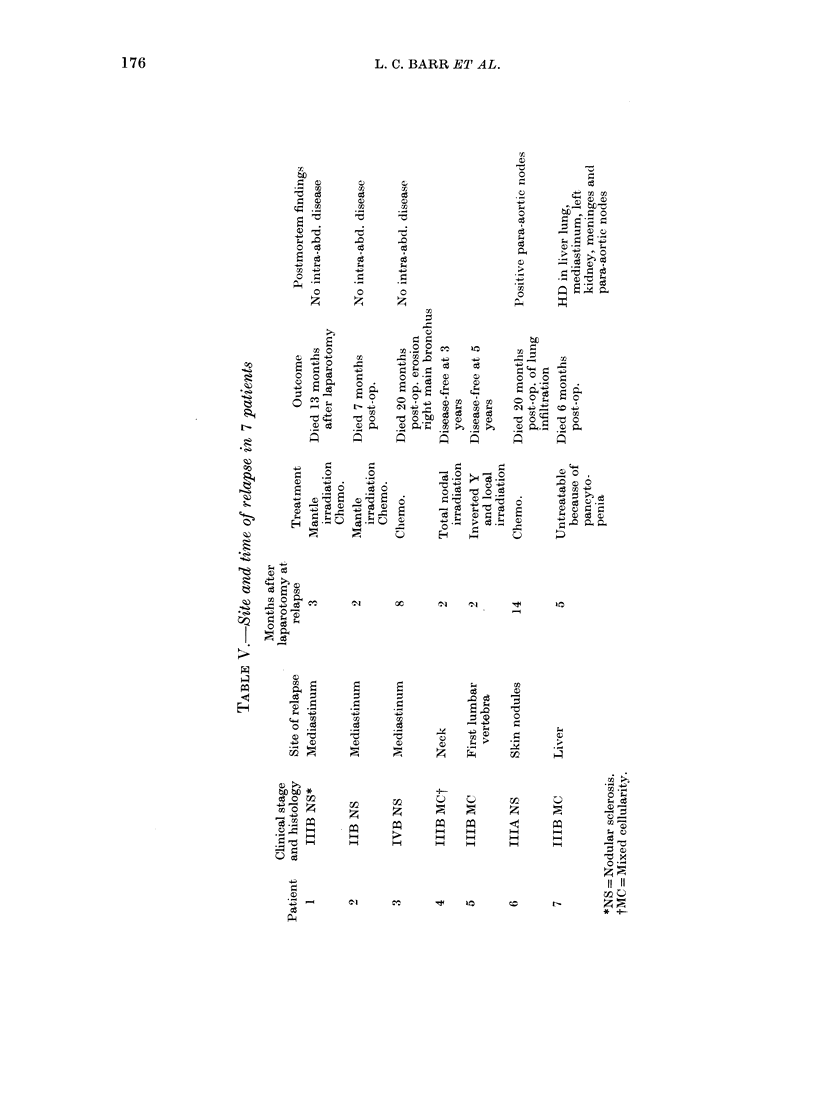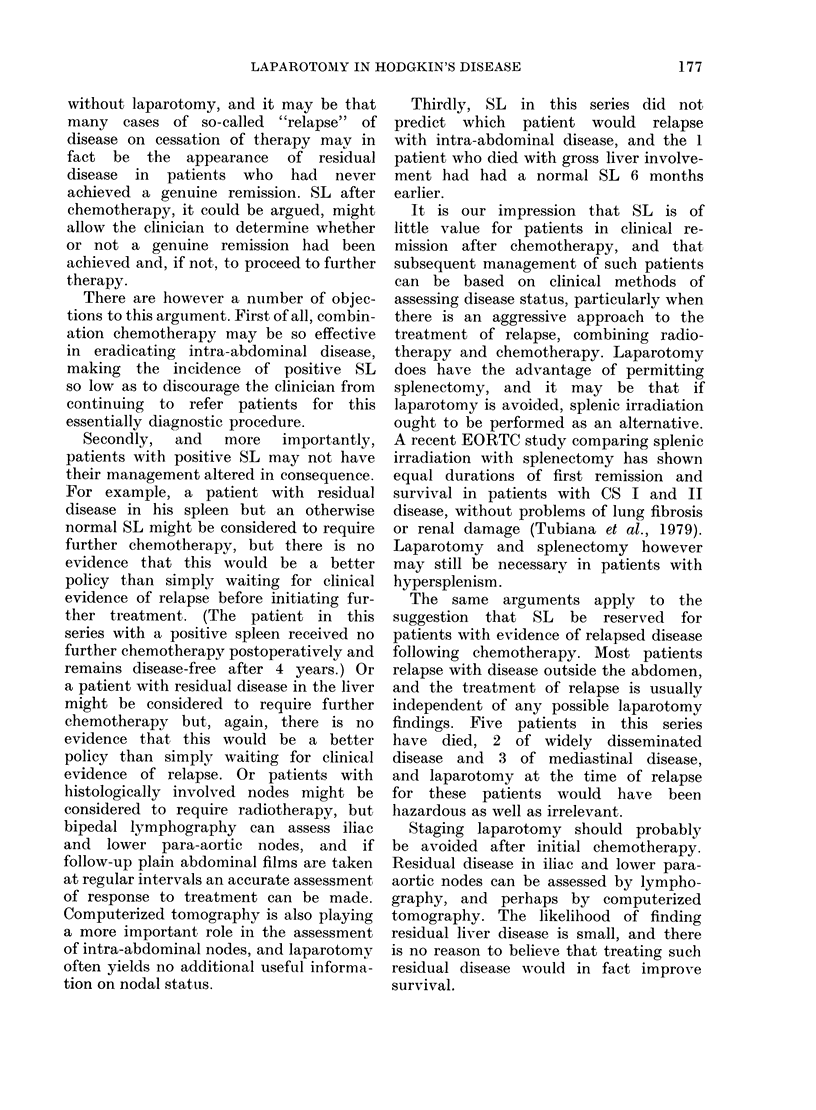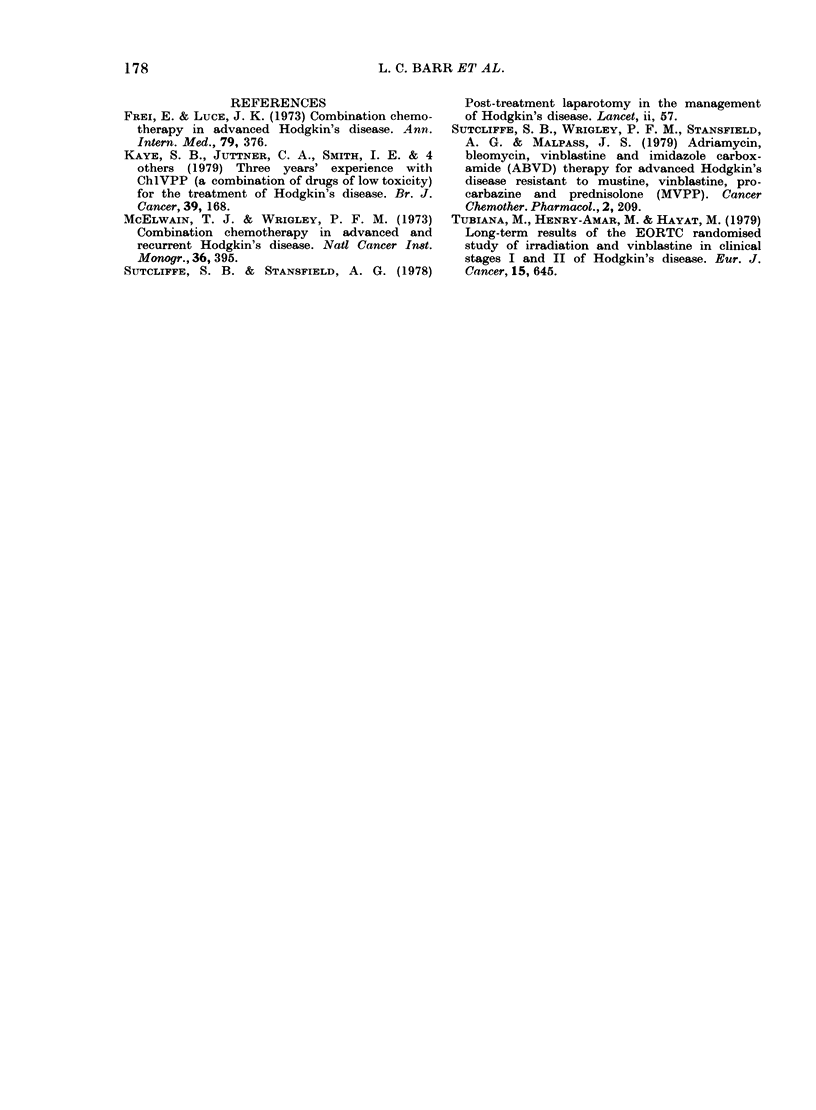# Postchemotherapy staging laparotomy in Hodgkin's disease.

**DOI:** 10.1038/bjc.1982.30

**Published:** 1982-02

**Authors:** L. C. Barr, J. P. Glees, T. J. McElwain, M. J. Peckham, J. C. Gazet

## Abstract

Seventeen patients with Hodgkin's disease who had a staging laparotomy (SL) within 2 months of the completion of initial chemotherapy are presented. Only 1 patient had a positive laparotomy. Postchemotherapy SL allows any residual active disease to be assessed, but the incidence of positive finding may be small, and such findings are unlikely to alter subsequent management. SL following chemotherapy is therefore not recommended either for patients in clinical remission or for patients with evidence of relapsed disease.


Br. J. Cancer (1982) 45, 174

### POSTCHEMOTHERAPY STAGING LAPAROTOMY IN HODGKIN'S DISEASE

L. C. BARR, J. P. GLEES, T. J. McELWAIN, M. J. PECKHAM AND J-C. GAZET

*From the Royal Marsden Hospital, Downs Road, Sutton, Surrey*

Received 23 June 1981 Accepted 23 October 1981

**Summary.—Seventeen patients with Hodgkin's disease who had a staging laparotomy (SL) within 2 months of the completion of initial chemotherapy are presented. Only 1 patient had a positive laparotomy. Postchemotherapy SL allows any residual active disease to be assessed, but the incidence of positive finding smay be small, and such findings are unlikely to alter subsequent management. SL following chemotherapy is therefore not recommended either for patients in clinical remission or for patients with evidence of relapsed disease.**

DURING THE LAST 5 years (September 1975 to August 1980) 17 patients with Hodgkin's disease (HD) have had a staging laparotomy (SL) under the care of one surgeon (J-C.G.) within 2 months of the completion of a variable number of courses of chemotherapy, given as initial treatment after diagnosis.

### PATIENTS

The indications for the initial chemotherapy are shown in Table I, and were usually that laparotomy was considered hazardous in view of extensive mediastinal disease. Laparotomy was thus postponed until the patient was considered to be in clinical remission. One patient however underwent laparotomy before he was thought to have achieved remission because he became thrombocytopenic after 3 courses of ChlVPP (Kaye *et al.*, 1979). Occasionally there were other factors that influenced the decision to postpone laparotomy (Table II).

TABLE I.—*Indications for chemotherapy*

| | |
|---|---|
| Superior vena caval obstruction | 3 |
| Bulky mediastinal disease | 9 |
| Bulky infradiaphragmatic disease | 1 |
| Lung infiltration | 3 |
| Treatment elsewhere for Stage IIIB disease | 1 |
| Total | 17 |

TABLE II.—*Other factors indicating initial chemotherapy*

| | No. of patients |
|---|---|
| Severe "B" symptoms | 2 |
| Severe osteoarthritis | 1 |
| Age (11 years) | 1 |

The clinical stages of the patients are shown in Table III. There were 6 Stage II patients (CS II), 5 with supradiaphragmatic disease alone and 1 with extensive infradiaphragmatic disease alone, including positive intra-abdominal nodes on lymphography.

There were 8 CS III patients, with mediastinal disease and positive lymphograms. Three patients were considered radiologically to have lung infiltration on presentation and were staged as CS IV. Fifteen patients had lymphography before chemotherapy, 11 of whom were positive.

The type of chemotherapy is shown in Table IV. Eleven patients had ChlVPP alone: 1 patient with 3 courses until laparotomy and splenectomy which was considered advisable in view of thrombocytopenia, and 10 with 4–8 courses until clinical remission

TABLE III.—*Clinical stage (CS) of 17 patients having laparotomy after initial chemotherapy*

| CS | No. of patients |
|---|---|
| IIA | 3 |
| IIB | 3 |
| IIIA | 2 |
| IIIB | 6 |
| IV | 3 |
| Total | 17 |

TABLE IV.—*Chemotherapy given before laparotomy*

| Chemotherapy | No. of patients |
|---|---|
| ChlVPP alone | 11 |
| ChlVPP+"second-line" | 3 |
| MOPP alone | 2 |
| MVPP+ChlVPP | 1 |
| Total | 17 |

was achieved. Three patients had ChlVPP followed by "second line" combination chemotherapy (MOPP (Frei & Luce, 1973) or ABVD (Sutcliffe *et al.*, 1979)) when remission was not achieved with ChlVPP alone. Three patients started chemotherapy with MOPP or MVPP (McElwain & Wrigley, 1973) at other hospitals before referral. Five patients had Mantle irradiation pre-operatively in addition. Sixteen of the 17 patients were therefore considered to be in remission at the time of laparotomy, which was performed to determine the presence of residual intra-abdominal disease.

### RESULTS

*Laparotomy findings*

Only one patient had a positive laparotomy, consisting of an involved spleen and porta hepatic node. He was a CS IIIB patient and had received 7 courses of ChlVPP. The patient who became thrombocytopenic after 3 courses of ChlVPP was found to have a normal spleen.

The yield of only 1 positive laparotomy is less than would be expected for a group of patients with untreated HD of similar clinical stages, as two-thirds of patients with HD and positive lymphograms are found to have involved spleens at laparotomy, *i.e.* in this study 1/11 patients with positive lymphograms had a positive spleen.

*Post-operative complications*

Two patients had postoperative complications (1 wound infection and 1 pelvic abscess) but this incidence of infective complications was no greater for SL in patients who have not received chemotherapy. The 11-year-old child received prophylactic penicillin, and remains disease-free after 5 years.

*Treatment after laparotomy*

Eight patients had no further treatment; 8 had radiotherapy postoperatively, including the patient with a positive spleen, who received total nodal irradiation; and 1 had further chemotherapy because he had only received 3 courses pre-operatively.

*Follow-up*

The patients have been followed up for 1–6 years, and a total of 7 patients have relapsed. The sites and time of relapse are shown in Table V.

Only one patient relapsed with intra-abdominal disease. He had normal liver histology at laparotomy, but 5 months later developed hepatomegaly and jaundice and was found to have a grossly involved liver at postmortem examination.

### DISCUSSION

Sutcliffe & Stansfield (1978) described 4 patients out of 19 considered to be in clinical remission after chemotherapy, who had evidence of active disease at laparotomy, most commonly in the spleen, and suggested that SL was valuable after chemotherapy because the need for further therapy could be assessed on the basis of the presence or absence of residual disease in the abdomen. Our own experience, however, with only 1 positive SL out of 16 patients considered to be in clinical remission after chemotherapy, raises the objection that the incidence of positive findings may be so reduced by chemotherapy that SL in such patients is no longer justifiable.

Methods of assessing splenic or hepatic involvement in HD, such as ultrasonography, isotope scanning, and computerized tomography are unreliable, and this makes the definition of clinical remission after chemotherapy difficult. Residual disease after chemotherapy in the spleen or liver may therefore remain undetected

TABLE V.—*Site and time of relapse in 7 patients*

| Patient | Clinical stage and histology | Site of relapse | Months after laparotomy at relapse | Treatment | Outcome | Postmortem findings |
|---|---|---|---|---|---|---|
| 1 | IIIB NS* | Mediastinum | 3 | Mantle irradiation Chemo. | Died 13 months after laparotomy | No intra-abd. disease |
| 2 | IIB NS | Mediastinum | 2 | Mantle irradiation Chemo. | Died 7 months post-op. | No intra-abd. disease |
| 3 | IVB NS | Mediastinum | 8 | Chemo. | Died 20 months post-op. erosion right main bronchus | No intra-abd. disease |
| 4 | IIIB MC† | Neck | 2 | Total nodal irradiation | Disease-free at 3 years | |
| 5 | IIIB MC | First lumbar vertebra | 2 | Inverted Y and local irradiation | Disease-free at 5 years | |
| 6 | IIIA NS | Skin nodules | 14 | Chemo. | Died 20 months post-op. of lung infiltration | Positive para-aortic nodes |
| 7 | IIIB MC | Liver | 5 | Untreatable because of pancyto-penia | Died 6 months post-op. | HD in liver lung, mediastinum, left kidney, meninges and para-aortic nodes |

*NS = Nodular sclerosis.
†MC = Mixed cellularity.

without laparotomy, and it may be that many cases of so-called "relapse" of disease on cessation of therapy may in fact be the appearance of residual disease in patients who had never achieved a genuine remission. SL after chemotherapy, it could be argued, might allow the clinician to determine whether or not a genuine remission had been achieved and, if not, to proceed to further therapy.

There are however a number of objections to this argument. First of all, combination chemotherapy may be so effective in eradicating intra-abdominal disease, making the incidence of positive SL so low as to discourage the clinician from continuing to refer patients for this essentially diagnostic procedure.

Secondly, and more importantly, patients with positive SL may not have their management altered in consequence. For example, a patient with residual disease in his spleen but an otherwise normal SL might be considered to require further chemotherapy, but there is no evidence that this would be a better policy than simply waiting for clinical evidence of relapse before initiating further treatment. (The patient in this series with a positive spleen received no further chemotherapy postoperatively and remains disease-free after 4 years.) Or a patient with residual disease in the liver might be considered to require further chemotherapy but, again, there is no evidence that this would be a better policy than simply waiting for clinical evidence of relapse. Or patients with histologically involved nodes might be considered to require radiotherapy, but bipedal lymphography can assess iliac and lower para-aortic nodes, and if follow-up plain abdominal films are taken at regular intervals an accurate assessment of response to treatment can be made. Computerized tomography is also playing a more important role in the assessment of intra-abdominal nodes, and laparotomy often yields no additional useful information on nodal status.

Thirdly, SL in this series did not predict which patient would relapse with intra-abdominal disease, and the 1 patient who died with gross liver involvement had had a normal SL 6 months earlier.

It is our impression that SL is of little value for patients in clinical remission after chemotherapy, and that subsequent management of such patients can be based on clinical methods of assessing disease status, particularly when there is an aggressive approach to the treatment of relapse, combining radiotherapy and chemotherapy. Laparotomy does have the advantage of permitting splenectomy, and it may be that if laparotomy is avoided, splenic irradiation ought to be performed as an alternative. A recent EORTC study comparing splenic irradiation with splenectomy has shown equal durations of first remission and survival in patients with CS I and II disease, without problems of lung fibrosis or renal damage (Tubiana *et al.*, 1979). Laparotomy and splenectomy however may still be necessary in patients with hypersplenism.

The same arguments apply to the suggestion that SL be reserved for patients with evidence of relapsed disease following chemotherapy. Most patients relapse with disease outside the abdomen, and the treatment of relapse is usually independent of any possible laparotomy findings. Five patients in this series have died, 2 of widely disseminated disease and 3 of mediastinal disease, and laparotomy at the time of relapse for these patients would have been hazardous as well as irrelevant.

Staging laparotomy should probably be avoided after initial chemotherapy. Residual disease in iliac and lower para-aortic nodes can be assessed by lymphography, and perhaps by computerized tomography. The likelihood of finding residual liver disease is small, and there is no reason to believe that treating such residual disease would in fact improve survival.

REFERENCES

FREI, E. & LUCE, J. K. (1973) Combination chemotherapy in advanced Hodgkin's disease. *Ann. Intern. Med.*, **79**, 376.

KAYE, S. B., JUTTNER, C. A., SMITH, I. E. & 4 others (1979) Three years' experience with ChlVPP (a combination of drugs of low toxicity) for the treatment of Hodgkin's disease. *Br. J. Cancer*, **39**, 168.

MCELWAIN, T. J. & WRIGLEY, P. F. M. (1973) Combination chemotherapy in advanced and recurrent Hodgkin's disease. *Natl Cancer Inst. Monogr.*, **36**, 395.

SUTCLIFFE, S. B. & STANSFIELD, A. G. (1978) Post-treatment laparotomy in the management of Hodgkin's disease. *Lancet*, ii, 57.

SUTCLIFFE, S. B., WRIGLEY, P. F. M., STANSFIELD, A. G. & MALPASS, J. S. (1979) Adriamycin, bleomycin, vinblastine and imidazole carboxamide (ABVD) therapy for advanced Hodgkin's disease resistant to mustine, vinblastine, procarbazine and prednisolone (MVPP). *Cancer Chemother. Pharmacol.*, **2**, 209.

TUBIANA, M., HENRY-AMAR, M. & HAYAT, M. (1979) Long-term results of the EORTC randomised study of irradiation and vinblastine in clinical stages I and II of Hodgkin's disease. *Eur. J. Cancer*, **15**, 645.